# From Print to Screen: Regulatory Considerations to Adopting Innovative Approaches for Patient Information and Safety

**DOI:** 10.1007/s43441-019-00018-0

**Published:** 2019-12-09

**Authors:** Winona Rei R. Bolislis, Charlie Mortazavi, Rossana Riccioni, Paul-Etienne Schaeffer, Thomas C. Kühler

**Affiliations:** 1 Sanofi, Chilly Mazarin, France; 2 Global Regulatory Affairs, Sanofi R&D, 1 Avenue Brossolette, 91385 Chilly Mazarin, France

**Keywords:** e-Label, Digital, Regulatory, Patient safety, PIL, Pharmacovigilance

## Abstract

Patient information leaflets (PILs) differ across regulatory jurisdictions—its form and structure are dependent on the regulations it conforms to. Yet, physical or paper-based documents remain to be the most prevalent way of delivering important information to patients. As technology continues to enhance our daily activities, patients are increasingly utilizing digital platforms to facilitate access to relevant product information, hence questioning the continuous viability of physical PILs. This paper aims to present the growing importance of transitioning from print to screen via dynamic electronic product information, as a way of expanding access and utility of patient information. It provides considerations or reflection points for regulators when adopting digital platforms to ensure that stakeholders, especially patients, receive trusted and real-time information on available and approved medicinal products. We underscore these with examples and case studies from countries and businesses that have adopted or are transitioning to such platforms.

## Introduction

A patient information leaflet (PIL) is a document that provides detailed information for patients or caregivers on the use, administration, risks, storage, and disposal of a medicine. It is commonly printed on paper and inserted in or attached to the product package. The information, structure, and layout vary across jurisdictions, based on a defined regulation (Refer to Annex 1).

Given that information on a printed piece of paper is static, any revisions needed to ensure the safe use and administration of a product must go through a lengthy process that is then only reflected in a newly printed version of the paper document. In addition, the need to guarantee patient safety has led to increasing regulations that require additional information and notices to be included in the PIL—often resulting in a lengthy document that is difficult to read by the patient or caregiver. For example, a recent study conducted in the United Kingdom found that 63% of the information in a paper PIL was considered difficult for older people to understand [1].

To date, paper-based leaflets have been the only formally recognized forms of delivering patient information. With the rapidly evolving technology and digital platforms available to it, why has the sector not transitioned?

Evidently, recent advancements in technology have transformed the way people access information. Between 2007 and 2017, global internet use grew from 20% of the total population to 49% [2]. At the end of 2018, 60% of the global population owned a smartphone and this is expected to further increase to 79% by 2025, primarily driven by mobile subscription growth in the Asia-Pacific region [3].

The internet serves as a rich source of health-related information. A survey conducted in Europe in 2014 across 26 566 respondents from different social and demographic groups found that 59% use the internet to search for health-related information on general health topics, specific diseases, and medical treatments and procedures [4]. Another survey conducted in 2017, including 1 509 individuals in the United States aged 18 and above, found that 57% of them accessed the internet first for health-related information compared to 32% who first sought information from doctors or physicians [5].

The shift from paper to dynamic electronic PILs or e-PILs is a first step to providing patients with the opportunity to receive up-to-date and clear information on a product and provide consumers with an avenue to regularly monitor a product’s characteristics as well as enable methods to improve readability and comprehension. Moreover, it can help facilitate communication in real-time across health care professionals, regulators, manufacturers, and patients on additional or updated information on the product.

For the purpose of this paper, we refer to *dynamic* e-PILs as patient information on a medical product, approved by a health authority, accessible electronically or online which have sections or information that can be opened in a screen and displayed according to the user’s preference. We qualify the term “dynamic” as e-PILs that allow for more flexibility on the presentation of its content, for example, adjustments to the format (e.g. font or image); integrated audio and video; search or dictionary functions; and any additional interactive functions to improve the presentation of information.

Adopting a dynamic e-PIL comes with various challenges, but can serve as a viable complement or solution to the difficulties faced by patients when relying on traditional paper package inserts [6]. One of the most immediate benefits of using e-labels, such as the e-PIL, is a considerable decrease in the time and resources needed to update and amend patient information, avoiding the possibility of having a marketed medicinal product with a dated package insert. The adoption of these platforms to deliver effective patient information therefore require bold changes: be it amendments to current legislation or the determination to adopt and deploy new and emerging technologies for the benefit of patients and society as a whole.

This commentary presents regulatory considerations that we believe are valuable for regulators to take into account when shifting to or adopting dynamic e-PILs and supports these with practical examples and case studies from literature reviews and the authors’ regulatory expertise and experiences.

## Regulatory Considerations for the Effective Adoption of Digital Platforms

### Promote a Digital Outlook and Act for Change

We believe that strong political commitment at the highest level is important in ensuring that society accepts the use of and transition to digital platforms. A clear endorsement and support to achieve this can help overcome administrative resistance and safeguard it from singular interests. For example, some international organizations and countries have developed clear guidelines and strategies that reflect their future outlook with regard to digital platforms (Box 1).

Box 1. Guidelines and Strategies for Digital Platform Adoption **International**

**WHO Recommendations on Digital Interventions for Health System Strengthening (2019)**

The guideline provides evidence-based recommendations when adopting digital health interventions, including its feasibility to relevant stakeholders and some considerations when implementing such transition or adoption [7].

**Country-specific**

**National Guidelines on On-Screen Display of Medicines Information (2017) and Australia’s National Digital Health Strategy (2018)**

In 2017, the Australian Commission on Safety and Quality in Health endorsed a set of guidelines for on-screen display of medicines information to ensure a standard design for the presentation of medicine information for continued patient safety. Following this, Australia introduced its National Digital Strategy in 2018, which underscored the importance of access to digital information. This strategy was drafted with the participation of a number of stakeholders across the country and endorsed by the local governments of Australia [8, 9].

**Denmark’s Digital Health Strategy (2018)**

Denmark’s National Digital Health strategy highlights the importance of digitization for patient care and treatment. The strategy, set for 2018–2022, includes clear support to apps that provide targeted patient information on a disease and endorses data-driven technologies to support in decision-making and prescribing of medicines. The strategy aims to have better coherence and greater geographical equality in the provision of health services. The strategy focuses on five main areas, including (1) the patient as an active partner; (2) knowledge on time; (3) prevention; (4) progress and common building blocks; and (5) trustworthy and secure data [10].

**Israel’s National Digital Health Program (2018)**

Following the National Digital Strategy introduced in 2017, the government of Israel has introduced the national digital health program as a way of increasing access and availability to health information and improve the overall health care system in Israel [11].

**Japan 2035 (2015)**

Japan has been among the pioneers in developing innovative healthcare. Introduced in 2015, the Japan 2035 vision aims to build and design a health care system for all members of society by 2035. Among others, this includes the need to improve the supporting infrastructure through technological development, including the development of health care databases to support telemedicine applications and a strong online database for disease management and treatments [12].

### Review and Streamline

Regular review is necessary to ensure that regulations are suited to the current technological developments; encourage innovation; and, more importantly, ensure that safe, efficacious, and quality medicinal products are received by the patients in a timely manner (Box 2).

Furthermore, overlapping regulations should be streamlined to ensure that any newly introduced or updated requirements do not contradict with existing standards at a national and, if applicable, at a regional level to ensure that there is a harmonized approach in the use of digital platforms.

Box 2. Providing Patients with Immediate Access to Best, Available Products Vaccines and consumer health care products could benefit from a review of regulations that favour a shift from paper-based product information leaflets to dynamic electronic patient information leaflets (e-PILs).

Medicine shortage, notably vaccines, is an important issue monitored by the entire health sector. The number and complexity of labelling requirements, such as country-specific labels and leaflets included in the product package, **can have an effect on** supply shortages [13]. This is despite the fact that most vaccines are administered through or with the oversight of highly educated and trained health care providers, without the need to thoroughly go through the physical PIL.

In terms of consumer health products or non-prescription medical products, consumers are often self-informed and seek the needed information online or will receive information or guidance at the pharmacy or local outlet levels. Information provided through digital media, instead of an inserted leaflet, therefore provides the possibility for consumers to have the best available updated information on a product to help facilitate self-selection, even before visiting the local shop or pharmacy.

### Set Minimum Requirements and Encourage Flexibility in Implementation

Countries may propose and set minimum requirements on the adoption of digital e-PILs or digital platforms in general. By setting minimum requirements, countries may adopt aligned principles without being overly prescriptive while ensuring patient adherence and safety. A set of proposed principles are presented in Box 3.

Contemporary or digital platforms provide the opportunity to increase product information utility and value-add; however, a flexible approach must be adopted when deciding to transition to dynamic e-PILs. Dynamic e-PILs can be introduced on a voluntary basis—considering them as a complement, rather than an ultimate alternative to physical PILs inserted in the package. Countries could adopt dynamic e-PILs as a *default* medium, and offer paper-based options as and when appropriate. This permits the possibility of transitioning towards paperless information, without undermining current rules and regulations (Box 4).

Staging the transition would be essential in allowing a certain period for countries to be sufficiently informed or master digital media. In 2017, the Asia-Pacific Economic Cooperation (APEC) came up with a set of best practices for electronic labelling and underscored various e-labelling policy development stages and recommended actions (Table 1).

**Table 1.** e-Labelling Policy Development Stages.

Policy Development Stage	Description
Status quo	Using physical labelling to demonstrate conformity
Develop	Build out initial e-labelling guideline and prepare for pilot and/or implementation
Pilot	Roll out limited, voluntary pilot and analyse results of initial policy
Finalize/implement	Incorporate results of analysis and stakeholder feedback; educate stakeholders of changes
Improve/build consensus	With domestic policy in place, work to align e-labelling approaches internationally

*Source:* APEC (2017). Best Practices for Electronic Labeling. Asia-Pacific Economic Cooperation Subcommittee on Standards and Conformance.

Box 3. Five Principles for the Adoption of Dynamic e-PILs **Principle 1:***Allow access to health authority approved product information through digital means*

Current labelling paradigms are dated and do not leverage the possibilities enabled by advances in digital technologies. Making product and labelling information available through the use of contemporary digital technologies (e.g. electronic devices, tools, systems, or platforms) would save time and increase the accuracy of the product information and improve safety. It would lower the risk of medicines and vaccines shortages and save thinly stretched resources for all stakeholders involved in the processes of furnishing PILs—be it regulators or sponsors.

**Principle 2:***Increase product information utility*

Digital technologies present the potential to introduce new features such as video or audio content, provide interactive (chatbot) services, or tailored “on-demand” services to increase product utility and improve patient value-add. This also facilitates patient engagement by providing an interactive, user-friendly, and customizable product information experience tailored to the treatment of their condition. Its ease-of-use can help drive compliance and uptake of a specific therapeutic intervention.

**Principle 3:***Stage the transition*—*offer paper-based product information when appropriate*

Paper prints may still be required for those not comfortable with, or sufficiently trained, to master digital media. The option to receive the most up-to-date official product information should remain guaranteed for these individuals. Digital media-based product information can therefore be the default option, but paper-based options should still be offered for all product categories when appropriate, possibly at pharmacy level. This will safeguard equal access to product information for all individuals independent of level of digital literacy.

**Principle 4:***Protect and secure data*

As information is increasingly processed online, personal data must benefit from the highest standard of privacy and protection and comply with a jurisdiction’s set data protection principles. Consent must be provided by the user for data to be collected and stored from electronic or digital services, such as information from product searches.

**Principle 5:***Measure process improvement*

Increasing improvements in technology provides opportunities to upgrade processes. A shift from paper to digital platforms is one step to facilitating access to and understanding of patient information. Monitoring and measuring process improvement in the transition to digital platforms is therefore important in evaluating the broader use of dynamic e-PILs and other functionalities.

Box 4. Examples of Online or On-Demand Patient Leaflets **Australia**

The Australian government has made printed patient information leaflets, locally known as consumer medicine information (CMI), optional as package inserts. Instead, patients can access the needed information digitally or, alternatively, this can be printed via the pharmacist or doctor. Patients can access product information and CMI on a registered prescription medicine via a mobile phone using the MedSearch™ app.

https://www.tga.gov.au/conumer-medicines-information-cmi

**Italy**

The Italian Drug Agency’s Drug Data Bank provides patients with up-to-date information on medicines. Companies are asked to provide the updated information to the database within 30 days of approval. Patients are allowed to have onsite printing through pharmacies.

https://farmaci.agenziafarmaco.gov.it/bancadatifarmaci/home

**Spain**

Spain’s Agency of Medicine and Medical Devices developed a Medicine Online Information Center that displays information on medicines, including leaflet information, in various formats including in sign language. The website also provides information on available medicines in community centres or pharmacy, as well as details referring to a product’s authorization and marketing status.

https://cima.aemps.es/cima/publico/home.html

**Sweden**

Fass.se is a trade-association online service that is updated daily and provides information on available medicines in the EU. It serves as a repository for summary of product characteristics and package leaflets approved by the Swedish Medical Products Agency and the EMA. It is regarded as one of the most consulted database on medicine information in the world, and is also accessible via a smartphone.

https://www.fass.se/LIF/startpage

**United States**

DailyMed is FDA’s official provider for trustworthy drugs marketed in the US, including information on package inserts. It provides standard, comprehensive, up-to-date, and downloadable content on the medical product and its respective inserts as well as manufacturing information intended to provide visibility into the supply chain for health care professionals and patients.

https://dailymed.nlm.nih.gov/dailymed

### Ensure Transparent Stakeholder Engagement and be Patient-Centric When Designing Platforms

Stakeholder engagement ensures that the adoption of new or updated regulations or platforms is effective and feasible. We refer to stakeholders as all relevant parties that should be involved, including but not limited to regulators, industry, health technology assessors, payers, health care professionals, and patients. Making ideas or projects transparent and open for discussion to stakeholders throughout the project helps in preparing stakeholders in meeting new requirements and processes.

Part of ensuring stakeholder engagement is embracing patient-centric approaches. Gaining insights and perspectives from patients and empowering them in the process would help transform dynamic e-PILs to their benefit, as it becomes designed to suit their needs and preferences.

For this reason, we believe that a PIL is considered effective when it is read, understood, retained, and adhered to by the patient or caregiver. While a physical PIL may provide a standard approach to presenting information, it may not provide the flexibility to ensure that the information included is suitable and effective to the reader. Numerous studies have been made to judge a patient’s reactions to the PIL’s structure, format, and content based on regulatory guidelines, and have found certain formats and structure often illegible to the reader [14–17] (Box 5).

While it is important to ensure that the information included in the e-PIL is suitable to the patient, other initiatives to improve digital health literacy or the patient’s understanding of the information online can also encourage more patient-centric approaches and foster patient engagement (Box 6).

Box 5. Why Patient-Centric Approaches? A study conducted by Herber et al. [17] explored the emotional reactions and behaviour of individuals towards package leaflets and found that participants used the term “being shocked” at the substantial amount of information presented in the patient information leaflet and that illegible fonts as well as complicated medical terms resulted in information being rejected or causing non-adherence, hence making the individual seek someone that could explain the information in plain language or leading them to simply access the information through a medical dictionary or online [16].

Another study done by Schwappach et al. [15] across 1000 participants aged 50 and above found that patients favoured coloured leaflets, brief and clear summaries, and general health tips in their patient leaflets [15]. This is not necessarily how current physical PILs are presented but can be easily achieved through a dynamic e-PIL.

Box 6. Examples of Online Health Literacy Platforms Around the World **Government-led**

**IC-Health (Europe)**

IC-Health is a European Commission-funded platform that aims to improve digital health literacy in Europe through massive open online courses presented in 8 different national languages and designed for different sub-populations (children, teenagers, and elderly) or disease populations.

https://ichealth.eu

**HealthHub (Singapore)**

HealthHub is Singapore’s integrated online, personalized platform for Singaporeans and permanent residents of all ages to access health records, locate health services and make appointments, get health-related content, search for terms, and learn more on how to achieve a healthy lifestyle. The platform provides tailored content such as individual program and action plans to monitor or manage personal health. Users can also earn points by sharing articles, events, and apps on social media which can be used for grocery bills and other deals from participating partners.

https://www.healthhub.sg/

**Business-led**

**Hello Doctor (Africa)**

Available in 10 African countries, the app provides the possibility for users to communicate with health care providers, get daily doctor-vetted health tips and advice, and access free health care information.

https://www.hellodoctor.co.za/

### Guarantee Access, Availability, and Security

Ensuring access and availability of the information and digital platform is important when transitioning to the dynamic e-PIL. This entails guaranteeing that the patients know how and where to find the needed information on the medical product, and creating sufficient awareness on the platform’s availability.

When it comes to accessing information, this can be made through a single country-specific platform such as DailyMed in the United States or Fass.se in Sweden or through the manufacturer’s website. Quick response (QR) codes and barcode scanning are increasingly becoming commonplace—allowing patients or consumers to link information quickly on a product’s information through a mobile phone. In 2018, the European Medicines Agency (EMA) introduced a set of general principles of acceptability and rules of procedure to encourage the use of mobile scanning and other technologies for this purpose [18].

While guaranteeing access is essential, it is beneficial for society to ensure that its citizens receive the best, updated information on a medicinal product, which is available. Therefore, guaranteeing security of the information and limiting the access to modifications between the regulator and the manufacturer must be considered.

### Support Common Frameworks and Standards

Ensuring that patients receive consistent and high-quality information regardless of regulatory jurisdiction is an important consideration in the adoption of the dynamic e-PIL. Varying or fragmented legal frameworks often impede the inter-operability of systems and may duplicate efforts leading to the inefficient use of resources.

## The Future of Patient Information

While this paper focuses on the transition and adoption of dynamic e-PILs, more tailored approaches to providing patient information are emerging. This includes increasing personalized services that are available 24/7 to guide and inform the patient on the safe use and administration of specific medicinal products. These enablers could provide for more personal and more relevant sharing of information on specific diseases or conditions. A few examples include virtual medical assistants and artificial intelligence-driven chatbots, which are increasingly becoming a resource for health information—using speech, text, audio, and video to check symptoms and connect with medical representatives, obtain product information, or receive regular reminders on medication or treatments. Setting up personal notifications tailored on individual circumstances, such as drug interactions, disease history, or child-bearing potential, could be enabled so that immediate information can be highlighted and received by the patient. As these chatbot services become increasingly used, the sector could learn from experience gained and even further improve content provided and its presentation (Box 7).

Furthermore, the increasing use of wearables and smart machines is transforming the way we monitor our health and make decisions on the medicinal products we use—whether it means adhering to its use, guaranteeing safety, or choosing the right product based on one’s health history.

Box 7. What are Chatbots? A chatbot is a software robot that can communicate with an individual, in natural language, through automated conversations. Chatbots normally operate using libraries or references linking keywords and questions; however, with recent advances in artificial intelligence (AI), chatbots are increasingly analysing and understanding messages transmitted through “machine learning”.

Several chatbots have already been developed: For example, a chatbot called “*Nina*” is used to promote sleep, tailored according to the responses from a set of questions. Nina is available via a website or through social media platforms. Available 24/7, this chatbot also provides information on an associated product that stimulates better sleep among adults [19].

Other available online chatbots educates patients about diabetes [20], provides information for people living with skin conditions (e.g. psoriasis or chronic urticaria) [21], and also provides training and self-assessment to sales representatives [22].

## Conclusion

In this paper, we presented regulatory considerations to help inform regulators when adopting the dynamic e-PIL—that is, transitioning from the traditional, common use of paper leaflet to the use of on-screen or digital platforms for more effective patient information on medicinal products, as a way of continuously guaranteeing patient safety. This transition to dynamic e-PILs or e-labelling is possible with strong political or top-down commitment, an efficient and progressive regulatory environment, a patient-oriented focus, and a harmonized and coordinated approach.

Indeed, an increasingly digital future is inevitable—society will continue to move towards the adoption of new technologies. Hence, as knowledge and development in these areas progresses, we must persevere to ensure that no patient gets left behind.

## Annex 1: Region-Specific Clinical Labelling Requirements for Secondary or Outer Packaging

**Table:**

	US	Japan	Canada	EU + Annex 13
Minimum labelling criteria				
Contact name				R
Contact address				R
Contact telephone				R
Name: shipper, importer, manufacturer				
Drug name		R	R	R
Strength/potency/dosage				R
Dosage form				R
Route of administration				R
Quantity of dosage units				R
Batch/lot	R	R	R	R
Trial reference code			R	R
Subject number				R
Investigator name/number				R
Directions for use				R
“Clinical trial use” phrase	R	R	R	R
Storage conditions		R	R	R
Expiry date/period of use			R	R
”Keep out of reach of children”				R
Handling/special precautions		R	R	
Manufacture date				
Country-specific language		R	R	R

*Note: R*—*required.* This table is an example of labelling requirements and refers to clinical labels only. This table is provided for informational purposes and is not intended to provide legal advice. Each company is responsible for determining compliance with specific laws, regulations, and guidances

*Source:* Smith-Gick J. Barnes N, Barone, R, et al. The Near-Term Viability and Benefits of e-Labels for Patients, Clinical Sites, and Sponsors. DIA Therapeutic Innovation and Regulatory Science. 2018;5 2(5): 537-545. 10.1177/2168479018765463
